# Image Superresolution Reconstruction via Granular Computing Clustering

**DOI:** 10.1155/2014/219636

**Published:** 2014-12-28

**Authors:** Hongbing Liu, Fan Zhang, Chang-an Wu, Jun Huang

**Affiliations:** School of Computer and Information Technology, Xinyang Normal University, Xinyang 464000, China

## Abstract

The problem of generating a superresolution (SR) image from a single low-resolution (LR) input image is addressed via granular computing clustering in the paper. Firstly, and the training images are regarded as SR image and partitioned into some SR patches, which are resized into LS patches, the training set is composed of the SR patches and the corresponding LR patches. Secondly, the granular computing (GrC) clustering is proposed by the hypersphere representation of granule and the fuzzy inclusion measure compounded by the operation between two granules. Thirdly, the granule set (GS) including hypersphere granules with different granularities is induced by GrC and used to form the relation between the LR image and the SR image by lasso. Experimental results showed that GrC achieved the least root mean square errors between the reconstructed SR image and the original image compared with bicubic interpolation, sparse representation, and NNLasso.

## 1. Introduction

In the field of image processing, SR images are usually desired for later image processing and analysis. Improvement of picture information for human interpretation and helping representation for automatic machine perception are two principal application areas [1]. The resolution of a digital image can be classified in many different ways: pixel resolution, spatial resolution, spectral resolution, temporal resolution, and radiometric resolution [2–5].

An image of *N* pixels height by *M* pixels width can have any resolution less than *N* lines per picture height. But when the pixel counts are referred to as resolution, the convention is to describe the pixel resolution with the set of two positive integer numbers, where the first number is the number of pixel columns (width) and the second is the number of pixel rows (height), for example, as 7680 by 6876. Another popular convention is to cite resolution as the total number of pixels in the image, typically given as number of megapixels, which can be calculated by multiplying pixel columns by pixel rows and dividing by one million. Other conventions include describing pixels per length unit or pixels per area unit, such as pixels per inch or per square inch. None of these pixel resolutions are true resolutions, but they are widely referred to as such; they serve as upper bounds on image resolution [2].

SR is a technique that constructs high-resolution (HR) images from several observed LR images, thereby increasing the high frequency components and removing the degradations caused by the imaging process of the LR camera. The basic idea behind SR is to combine the nonredundant information contained in multiple LR frames to generate a HR image. A closely related technique with SR is the single image interpolation approach, which can be also used to increase the image size [1, 5]. However, since there is no additional information provided, the quality of the single image interpolation is very much limited due to the ill-posed nature of the problem, and the lost frequency components cannot be recovered. In the SR setting, however, multiple LR observations are available for reconstruction, making the problem better constrained. The nonredundant information contained in these LR images is typically introduced by subpixel shifts between them. These subpixel shifts may occur due to uncontrolled motions between the imaging system and scene, for example, movements of objects, or due to controlled motions, for example, the satellite imaging system orbiting the earth with predefined speed and path [6, 7].

GrC is a transformation method between the universe and the parts and is widely used in pattern recognition, information system, and so forth. Zadeh identified three fundamental concepts of the human cognition process, namely, granulation, organization, and causation [8, 9]. Granulation is a process that decomposes a universe into parts. Conversely, organization is a process that integrates parts into a universe by introducing operation between two granules. Causation involves the association of causes and effects. The construction of granule set, the operations between two granules, and the inclusion measure between two granules are main researches in GrC. In general, the fuzzy inclusion measure is induced by granule and union granule, such that the positive valuation functions of granules are used to define the fuzzy inclusion measure [10–15].

The present work uses GrC clustering to construct the SR image of the original image. The granules are represented as the hypersphere, and the join operation and meet operation are designed to obtain GS including the granules with different granularities. The fuzzy inclusion measures are compounded by the positive valuation functions.

The rest of this paper is presented as follows. The theoretical background of GrC clustering is described in Section 2.  Section 3 designs the GrC clustering algorithms. The image SR reconstruction experiments are used to demonstrate GrC clustering in Section 4.  Section 5 summarizes the contribution of our work and presents future work plans.

## 2. Theoretical Background

GrC clustering is discussed on the basis of the contribution of Kaburlasos and his colleagues in the view of set theory [10–15].

### 2.1. Representation of Granules

A granule is represented as a subset of *S* which is composed by the data with the similar features, and the size of granule is measured by the granularity defined by the maximal distance between data belonging to the same granule. In order to facilitate the study of granular computing, such as the operations between two granules, the granules are represented as the standard form, for example, the granule with the shape of circle in 2-dimensional space and the shape of hypersphere in *N*-dimensional space.

A granule is represented as the hypersphere **G** = (**C**, *R*), where **C** is the center of granule and *R* is radius of granule, and refers to the granularity of granule **G** which is measured by the maximal distance between center and the data included in granule. Particularly, a point **x** is represented by an atomic granule with the center **x** and granularity 0 in *N*-dimensional space. The distance between center **C** = (*c*_1_, *c*_2_,…, *c*_*N*_) and datum **x** = (*x*_1_, *x*_2_,…, *x*_*N*_) can be defined as follows:

(1) dx,C=x1−c12+x2−c22+⋯+xN−cN21/2.

### 2.2. Operations between Two Granules

The operations between two granules reflect the transformation between the macro world and the microscopic world of human cognition. When a person wants to observe the object more carefully, the object is partitioned into some suitable subobjects; namely, the universe is partitioned into some parts in order to study the object in detail in the view of the microscopic world. Conversely, if some objects have the same attributes, we regard them as a universe in the view of the macro world. The operations between two granules are designed to realize the transformation between the macro world and the microscopic world. Set-based models of granular structures are special cases of lattice-based models, where the lattice join operation ∨ coincides with set union operation ∪ and lattice meet operation ∧ coincides with set intersection operation ∩.

Join operation ∨ and meet operation ∧ are used to realize the transformation between the macro world and the microscopic world. Operation ∨ unites the granules with small granularities to the granules with the large granularities. Inversely, operation ∧ divides the granules with large granularities into the granules with small granularities. Join operation is associated with the dilation operation of mathematical morphology (MM), and meet operation is associated with the erosion operation of MM [15]. In image analysis field, dilation operation replaces all the pixel values in the neighborhood with the maximal pixel value, and erosion operation replaces all the pixel values in the neighborhood with the minimal pixel value [16]. Join operation ∨ and meet operation ∧ are designed as follows.

All points are regarded as atomic granules which are indivisible, and the join process is the key to obtain the larger granules compared with atomic granules. Likewise, the whole space is a granule with the maximal granularity, and the meet process produces the smaller granules compared with original granules.

For two hypersphere granules **G**_1_ = (**C**_1_, *R*_1_) and **G**_2_ = (**C**_2_, *R*_2_) in *N*-dimensional space, the join hypersphere granule is

(2) $$\begin{array}{c} G=G_{1}\vee G_{2}=\left(C,R\right). \end{array}$$

The center **C** of **G** and the granularity of **G** are computed as follows.

Firstly, the vector from **C**_1_ to **C**_2_ and vector from **C**_2_ to **C**_1_ are computed. If **C**_1_ = **C**_2_, then **C**_12_ = 0 and **C**_21_ = 0. If **C**_1_ ≠ **C**_2_, then **C**_12_ = (**C**_2_ − **C**_1_)/*d*(**C**_1_, **C**_2_) and **C**_21_ = (**C**_1_ − **C**_2_)/*d*(**C**_2_, **C**_1_).

**Figure 1:**
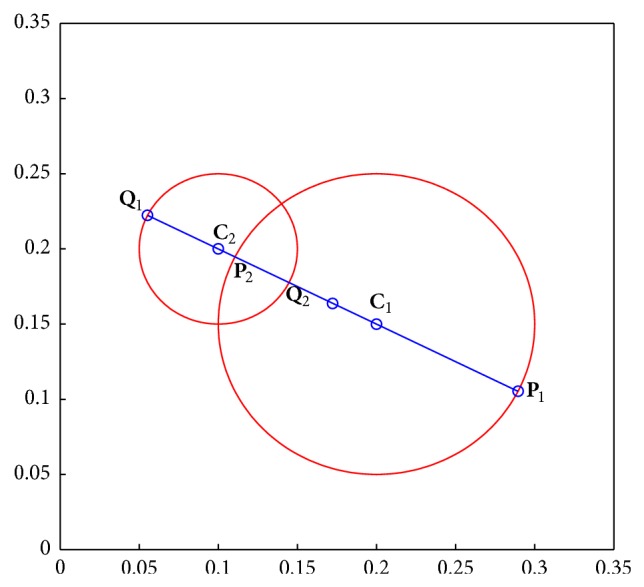
The cross-points between hypersphere granules and the line through **C**_12_. **P**_1_ and **P**_2_ are the cross-points between hypersphere **G**_1_ = (**C**_1_, *R*_1_) and the line through **C**_1_ and **C**_2_, and **Q**_1_ and **Q**_2_ are the cross-points between hypersphere **G**_2_ = (**C**_2_, *R*_2_) and the line through **C**_1_ and **C**_2_.

Secondly, the cross-points between the hypersphere **G**_1_ and the line through **C**_12_ are **P**_1_ = **C**_1_ − **C**_12_ *R*_1_ and **P**_2_ = **C**_1_ + **C**_12_ *R*_1_. The cross-points between the hypersphere **G**_2_ and the line through **C**_12_ are **Q**_1_ = **C**_2_ − *R*_2_ **C**_21_ and **Q**_2_ = **C**_2_ + *R*_2_ **C**_21_. The cross-points are shown in Figure 1.

Thirdly, the join hypersphere granule **G** is computed by the following formulas:

(3) G=G1∨G2=C=C1,  R=R1 if  R1≥R2,  dC1,C2≤R1−R2C=C2,  R=R2 if  R1<R2,  dC1,C2≤R2−R1C=0.5P1+Q1,  R=0.5dP1,Q1 if  dC1,C2>R1−R2.

Similarly, the meet hypersphere granule is computed:

(4) g=G1∧G2=⌀ if  dC1,C2>R1+R2c=C2,  r=R2 if  dC1,C2<R1−R2, R1≥R2c=C1,  r=R1 if  dC1,C2<R2−R1, R2≥R1c=0.5P2+Q2,  r=0.5dP2,Q2 if  dC1,C2>R1−R2.

### 2.3. Fuzzy Inclusion Measure

As mentioned above, for all **G**_1_, **G**_2_ ∈ GS, **G**_1_⊆**G**_1_∨**G**_2_, and **G**_2_⊆**G**_1_∨**G**_2_, **G**_1_∧**G**_2_⊆**G**_1_ and **G**_1_∧**G**_2_⊆**G**_2_. Namely, the operations between granule **G**_1_ and granule **G**_2_ are corresponding to the inclusion relation between granules **G**_1_ and **G**_2_:

(5) G1G2⟺G1∨G2=G2,G1∧G2G1.

The inclusion relation between two hypersphere granules is induced by the operations between two hypersphere granules.

The join hypersphere granule and the meet hypersphere granule are used to measure the fuzzy inclusion relation. The granularity *R* is used to define the fuzzy inclusion measure:

(6a) KG1,G2vG2vG1∨G2,

(6b) SG1,G2=vG1∧G2vG2,

where *v*(**G**) is the positive valuation function defined by Kaburlasos et al., which can be the linear function or nonlinear function [10–13]. A valuation function *v*: *L* → *R* is a mapping between a lattice *L* and a real number. The valuation function satisfies *v*(*a*) + *v*(*b*) = *v*(*a*∧*b*) + *v*(*a*∨*b*), *a*, *b* ∈ *L*. A valuation function is called positive if and only if *a* < *b*⇒*v*(*a*) < *v*(*b*) [14].

The hypersphere granule set is a mathematical lattice if the inclusion measure is defined as (6a) and (6b). More specifically, (6a) and (6b) can be used for hyperspheres based on the lattice of intervals on the line defined by the centers **C**_1_ and **C**_2_ of the hyperspheres (**C**_1_, *R*_1_) and (**C**_2_, *R*_2_), respectively, as explained in Example 2.8 in [11].

According to [15], the strictly increasing function is a positive valuation. For **G** = (**C**, *R*),

(7) $$\begin{array}{c} v\left(G\right)=R+\beta \end{array}$$

is a positive valuation function defined on GS, where *β* is a constant.

### 2.4. Fuzzy Algebraic Structures

For a training set *S* = {**x**_*i*_∣*i* = 1,2,…, *n*}, every datum *x*_*i*_ is represented as an atomic hypersphere granule which is indivisible, and the granule set is obtained. For the positive valuation function (7), the fuzzy inclusion relation between two hypersphere granules is computed by formulas (6a) and (6b). So the fuzzy algebraic structures 〈GS, *K*(**G**_1_, **G**_2_)〉 and 〈GS, *S*(**G**_1_, **G**_2_)〉 are formed by GS and *K*(**G**_1_, **G**_2_), where *K*(**G**_1_, **G**_2_) implies the operation between two hypersphere granules and *S*(**G**_1_, **G**_2_) implies the meet operation between two hypersphere granules. 〈GS, *K*(**G**_1_, **G**_2_)〉 and 〈GS, *S*(**G**_1_, **G**_2_)〉 are proved as fuzzy lattice, and *K*(**G**_1_, **G**_2_) and *S*(**G**_1_, **G**_2_) are fuzzy inclusion measures, which satisfied the following four conditions [12, 14, 17].

1. If **G** ≠ *⌀*, then *K*(**G**, *⌀*) = 0, *S*(**G**, *⌀*) = 0.
2. For **G** ∈ GS, *K*(**G**, **G**) = 1, *S*(**G**, **G**) = 1.
3. If **G**_1_ ≤ **G**_2_, then *K*(**G**, **G**_1_) ≤ *K*(**G**, **G**_2_)  *S*(**G**, **G**_1_) ≤ *S*(**G**, **G**_2_).
4. If **G**_1_∧**G**_2_ < **G**_1_, then *K*(**G**_1_, **G**_2_) < 1.

## 3. GrC Clustering

For the data set *S* = {**x**_*i*_∣*i* = 1,2,…, *n*} in *N*-dimensional space, we form the following three algorithms based on the aforementioned theoretical background.

**Figure 2:**
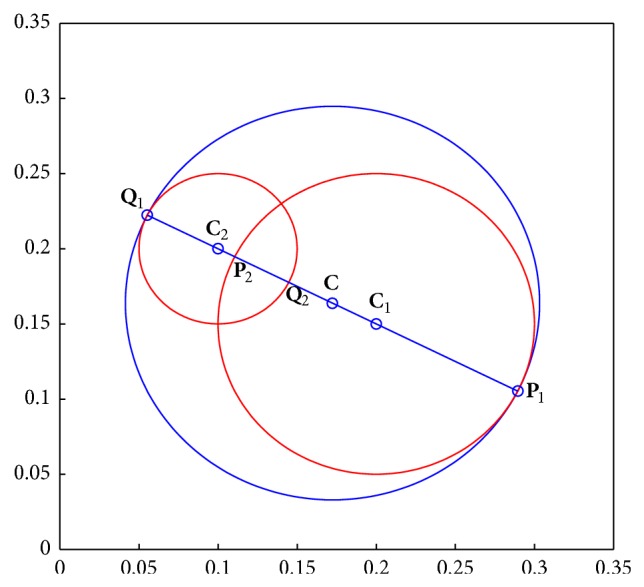
The join hypersphere granule between two hypersphere granules. The hypersphere with blue curve is the join hypersphere between two hyperspheres with red curves.

Algorithm 1 is the join process between two hypersphere granules and produces the hypersphere granule with the larger granularity compared with the original hypersphere granules. For example, the join hypersphere of hypersphere granules **G**_1_ = [0.2, 0.15, 0.1] and **G**_2_ = [0.1, 0.2, 0.05] in 2-dimensional space is **G** = [0.1724, 0.1638, 0.1309] as shown in Figure 2.

**Figure 3:**
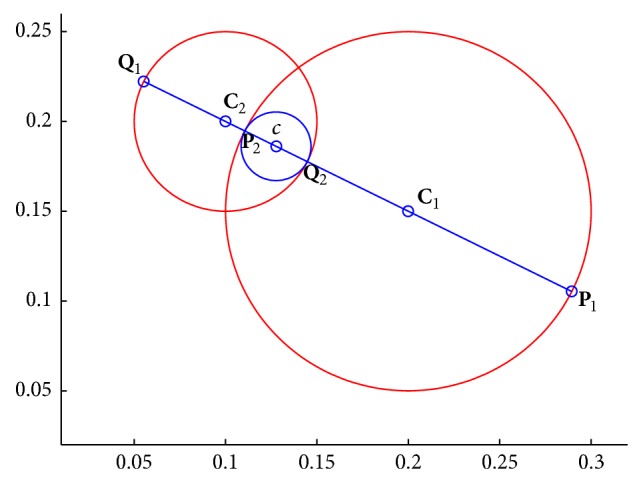
The meet hypersphere granule between two hypersphere granules. The hypersphere with blue curve is the meet hypersphere between two hyperspheres with red curves.

Algorithm 2 is the meet process between two hypersphere granules and produces the hypersphere granule with the smaller granularity compared with the original hypersphere granules. The meet process of hypersphere granule **G**_1_ = [0.2, 0.15, 0.1] and hypersphere granule **G**_2_ = [0.1, 0.2, 0.0.05] is *g* = [0.1276, 0.1862, 0.0191] as shown in Figure 3.

For data set *S*, the GrC clustering algorithms are proposed based on the join process by the following steps. Firstly, the samples are used to form the atomic granule. Secondly, the threshold of granularity is introduced to conditionally unite the atomic granules by the aforementioned join operation, and the granule set is composed of all the join granules. Thirdly, if all atomic granules are included in the granules of GS, the join process is terminated; otherwise, the second process is continued. The GrC clustering algorithms are described as in Algorithm 3.

**Figure 4:**
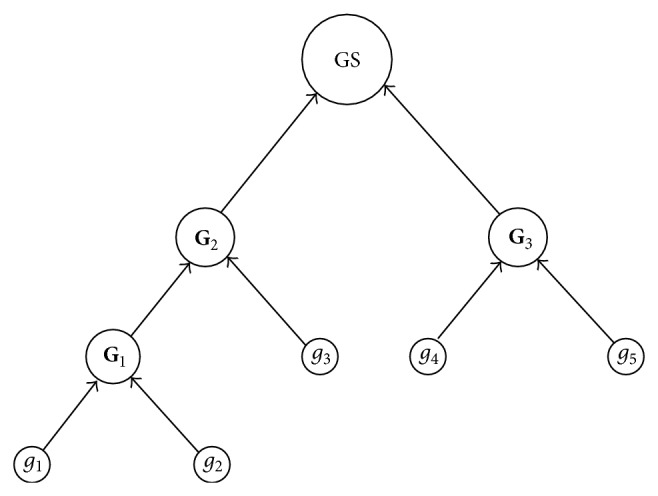
The clustering process of *TS* including 5 samples. Leafs denote the atomic hypersphere granules, branch points denote the join hypersphere granule, and the tree root denotes GS.

Suppose the atomic granules induced by *S* are *g*_1_, *g*_2_, *g*_3_, *g*_4_, and *g*_5_. The GrC clustering process can be described as the tree structure shown in Figure 4, leafs denote the atomic granules, root denotes GS including its child nodes **G**_2_ and **G**_3_, **G**_1_ is induced by join operation of child nodes *g*_1_ and *g*_2_, **G**_2_ is the join granule of **G**_1_ and *g*_3_, and **G**_3_ is the join granule of *g*_4_ and *g*_5_. The whole process of obtaining GS is the bottle-up process.

## 4. Experiments

Experimental settings used the same parameters in [18]; namely, the superresolution image is magnified by the input image with a factor of 3; for the low-resolution images, 3 × 3 low-resolution patches with overlap of 1 pixel between adjacent patches and the corresponding 9 × 9 patches with overlap of 3 pixels for the superresolution patches are used in our experiments. The experiments include three stages: sampling, training, and reconstruction.

The sampling stage is the generation of training set for the training images in [18]. In general, the training images are SR image. The purpose of sampling stage is to form the corresponding LR image of SR image. For color images, the illuminance component is applied to the proposed algorithms since humans are more sensitive to illuminance changes. Firstly, the color image is transformed into the gray image. Secondly, LR images are extracted from SR images, SR image patches and the corresponding LR image patches are selected to form the vector, and all the vectors are used to generate the training set. 91 training images are used to form the patches to train the granule set, and 999910 patches are extracted to form the training set S, which is redundant and has many of similar data.

**Figure 5:**
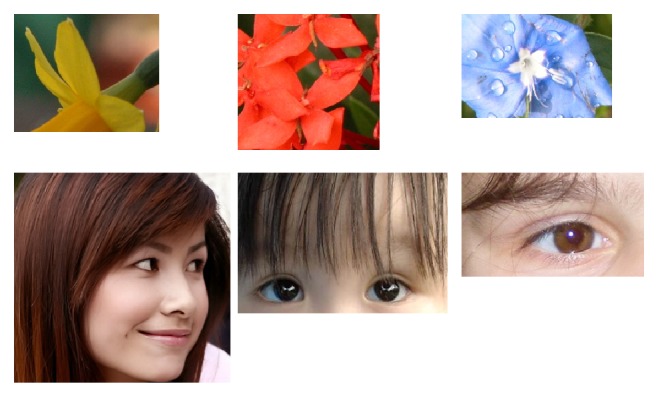
Training images with different sizes.

**Figure 6:**
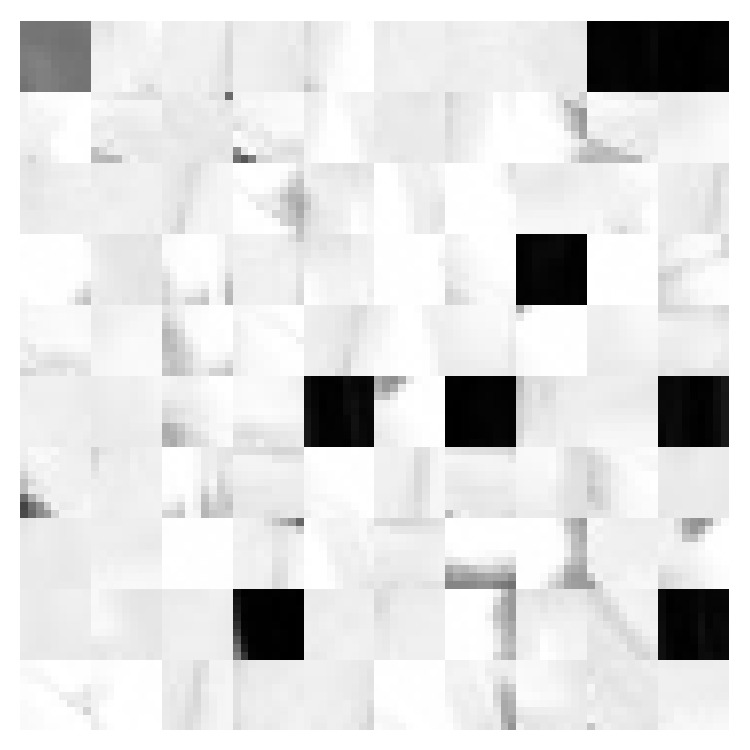
The patches sampled by GrC with *ρ* = 100 from the training image shown in Figure 5.

The training stage is to reduce the redundancy of training set by the aforementioned GrC clustering.  Figure 5 shows six training images with different sizes, such as flowers and faces, and the training set including redundancy patches is generated by the sampling stage.  Figure 6 shows the image patches trained by GrC clustering with *ρ* = 100.

The same reconstruction strategy as [18] is used to form the SR image in reconstruction stage. We compare SR image reconstruction via GrC clustering with bicubic interpolation [19], sparse representation [18], and NNLasso [20]. The performance included the SR reconstruction images and the RMSE between the SR reconstruction image and the original superresolution image.

**Figure 7:**
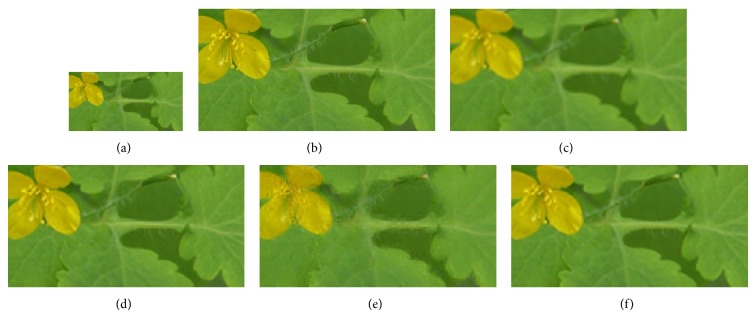
The flower image magnified by a factor of 3. Left to right: (a) low-resolution image, (b) the original image, (c) superresolution image by bicubic interpolation, (d) superresolution image by sparse representation, (e) superresolution image by NNLasso, and (f) superresolution image by GrC.

**Figure 8:**
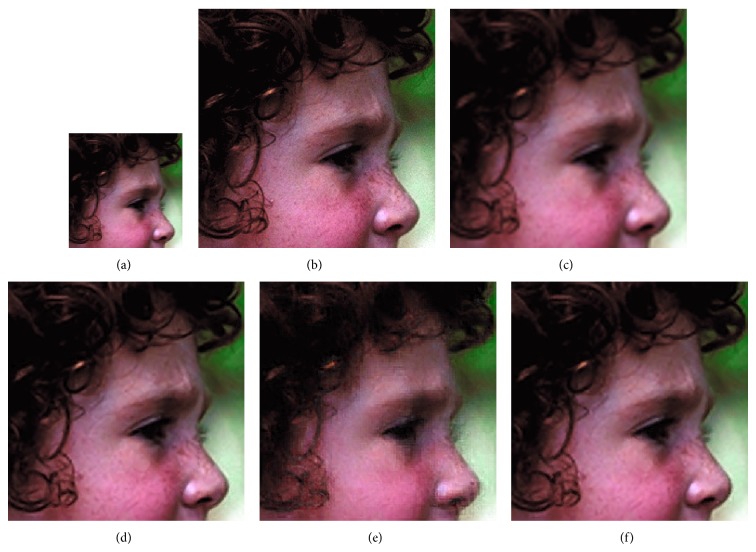
The girl image magnified by a factor of 3. (a) Low-resolution image, (b) the original image, (c) superresolution image by bicubic interpolation, (d) superresolution image by sparse representation, (e) superresolution image by NNLasso, and (f) superresolution image by GrC.

**Figure 9:**
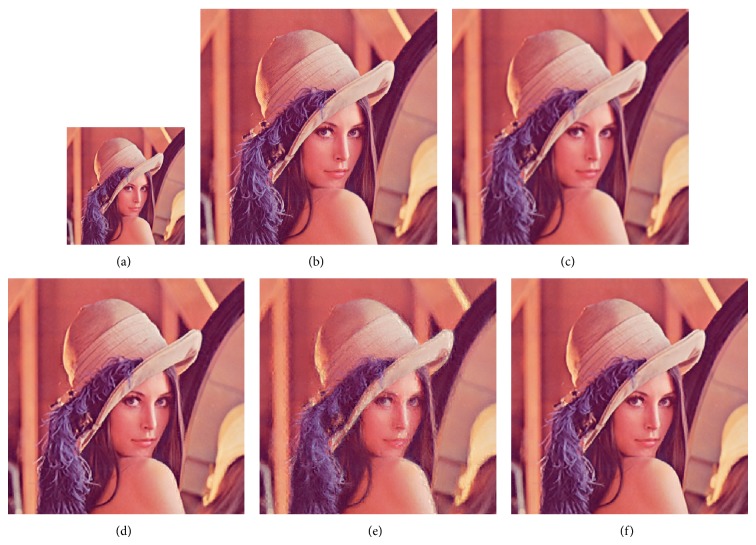
The Lenna image magnified by a factor of 3. (a) Low-resolution image, (b) the original image, (c) superresolution image by bicubic interpolation, (d) superresolution image by sparse representation, (e) superresolution image by NNLasso, and (f) superresolution image by GrC.

**Figure 10:**
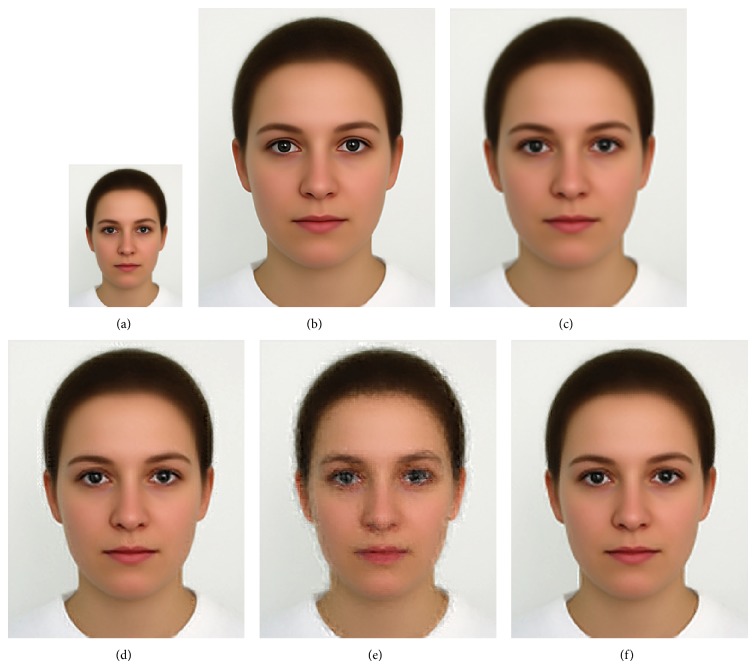
The average female face image magnified by a factor of 3. (a) Low-resolution image, (b) the original image, (c) superresolution image by bicubic interpolation, (d) superresolution image by sparse representation, (e) superresolution image by NNLasso, and (f) superresolution image by GrC.

**Figure 11:**
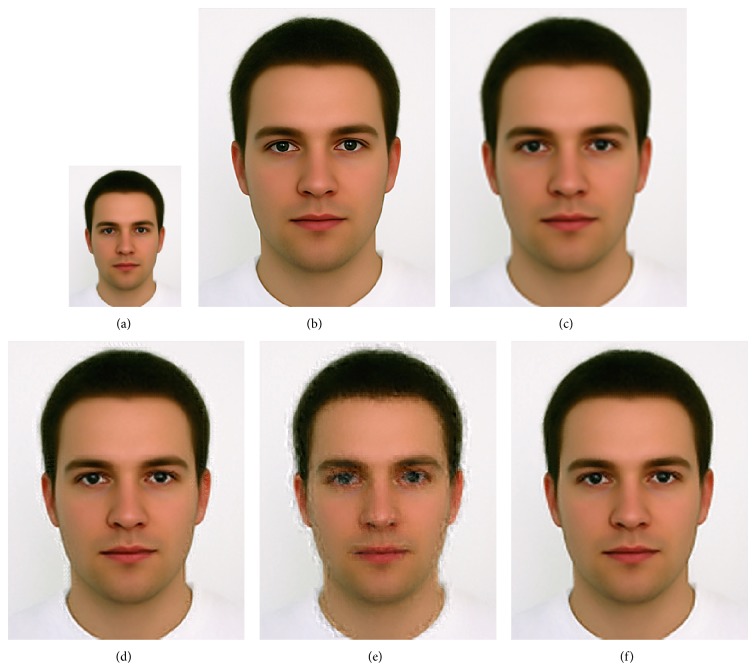
The average male face image magnified by a factor of 3. (a) Low-resolution image, (b) the original image, (c) superresolution image by bicubic interpolation, (d) superresolution image by sparse representation, (e) superresolution image by NNLasso, and (f) superresolution image by GrC.

We compared GrC clustering with sparse representation, bicubic interpolation, and NNLasso, on five test images of a flower [18], girl [18], Lenna [21], average female face [22], and average male face [22]. Firstly, training set including 999910 image patches is obtained in the sampling stage, and the redundancy of training set is reduced by GrC and sparse representation. Secondly, the LS images of testing images are resized by nearest method. Thirdly, the SR images are obtained by sparse representation, bicubic interpolation, NNLasso, and GrC clustering. The root mean square error (RMSE) between the superresolution images and the original images is listed in Table 1. From the table, we can see that the superresolution images by GrC are better than the superresolution by bicubic interpolation (bicubic), sparse representation (sparse), and NNLasso. The LS images, original images, and SR images are shown in Figures 7, 8, 9, 10, and 11. For human visual, the original images are the most clear, and the reconstruction images by NNLasso are blurry.

## 5. Discussion

The experimental results of the previous section demonstrate the effectiveness of image superresolution reconstruction via GrC. However, one of the most important questions for future investigation is to determine, in terms of the within-category variation, the number of raw sample patches required to generate a dictionary satisfying GrC. Because GrC is an online learning algorithm, the achieved granule set is related to the rank of training set. Image magnified by a factor of 3 is performed in the paper, and the larger magnification factors will increase the complexity of GrC and be discussed in the future works.

## Figures and Tables

**Algorithm 1:**
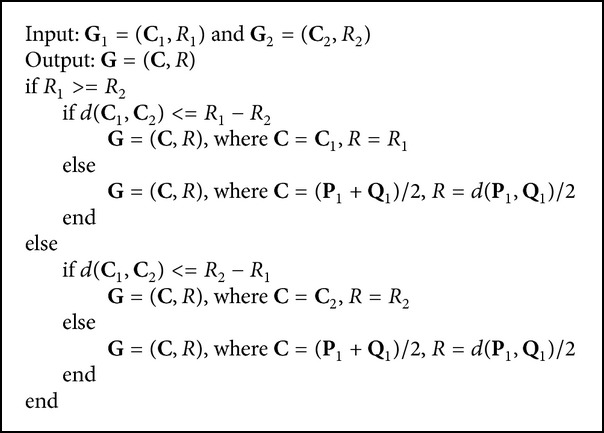
Computing **C** and *R* of join hypersphere granule **G** between **G**_1_ and **G**_2_.

**Algorithm 2:**
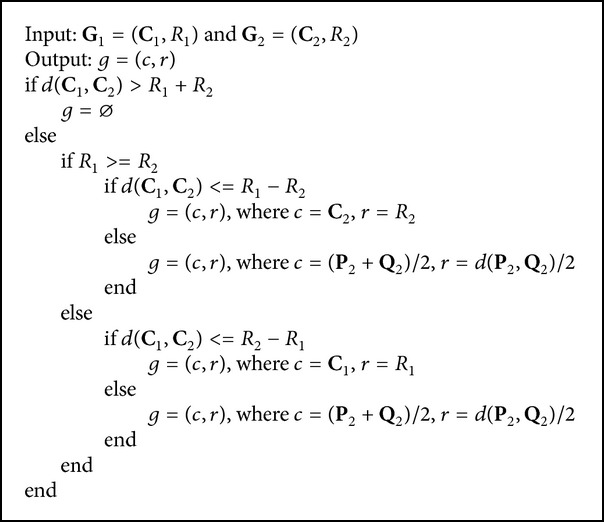
Computing *c* and *r* of meet hypersphere granule *g* between **G**_1_ and **G**_2_.

**Algorithm 3:**
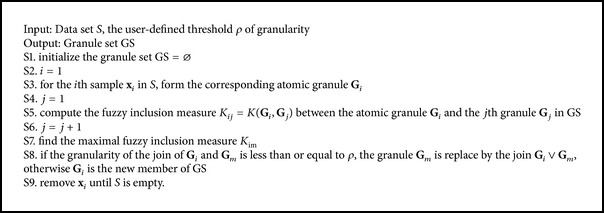
GrC clustering process.

**Table 1:** The RMSE of different methods for superresolution with magnification factor 3 with respect to the original images.

Images	Bicubic [19]	Sparse [18]	NNLasso [20]	GrC clustering
Flower (330 × 171)	4.0837	3.9240	6.5283	3.9153 (*ρ* = 0.7)
Girl (255 × 258)	6.8506	6.6383	9.9462	6.6318 (*ρ* = 0.7)
Lenna (512 × 512)	7.3515	6.9950	12.1007	6.9076 (*ρ* = 0.7)
Female face (400 × 320)	4.9347	4.7763	8.8875	4.4427 (*ρ* = 0.65)
Male face (400 × 320)	6.0613	5.7385	10.0067	5.3204 (*ρ* = 0.65)
